# Vaccination and Control Methods of West Nile Virus Infection in Equids and Humans

**DOI:** 10.3390/vaccines12050485

**Published:** 2024-05-01

**Authors:** Parker M. Cendejas, Alan G. Goodman

**Affiliations:** 1 Doctor of Veterinary Medicine Graduate Program, Washington State University, Pullman, WA 99164, USA; 2 School of Molecular Biosciences, Washington State University, Pullman, WA 99164, USA; 3 Paul G. Allen School of Global Health, College of Veterinary Medicine, Washington State University, Pullman, WA 99164, USA

**Keywords:** horse, vaccine, transgenic, paratransgenic, Swiss cheese

## Abstract

West Nile virus (WNV) is capable of causing severe neurologic disease in both humans and equines, making it a disease of importance in both human medicine and veterinary medicine. No targeted treatments exist for WNV infection in either humans or equines. Infection is treated symptomatically through management of symptoms like fever and seizures. As treatment for WNV is purely supportive, the response to WNV has focused primarily on methods of disease prevention. To this end, research efforts have yielded several effective vaccines for equine use as well as numerous conventional mosquito control techniques. Even with the implementation of these techniques, disease caused by WNV remains a concern since no human vaccine exists. Due to the lack of a human vaccine, novel preventative strategies are under active research and development. Of these strategies, some of the most conceptually promising are techniques using genetically modified mosquitoes, addressing the disease at the vector level with minimal ecological side effects. Taken together, the use of combined, synergistic methods, such as physical barriers, transgenic mosquitoes, and immunological targets, will be the best way to prevent WNV disease.

## 1. Introduction

West Nile virus (WNV) is an arthropod-borne virus (arbovirus) of the *Flaviviridae* family transmitted primarily by mosquitoes of the genus *Culex* [1]. It is a disease of worldwide health concern, with WNV now being endemic to the contiguous United States as well as much of Africa, Europe, West Asia, and Australia [2]. In the United States, WNV is the most common mosquito-borne disease [3]. Despite the increasing exposure of populations to mosquito-borne flaviviruses, such as WNV, and the expanding ranges of vector mosquitoes [4], there are limited resources to prevent and treat WNV infections. Accordingly, there is a significant need for new control and treatment options. As a vector-borne disease of significance in both human and veterinary medicine, the response to WNV has necessitated interdisciplinary collaboration, as exemplified by the utility of the One Health concept, which combines the fields of ecology, human health, and animal health. In this narrative review directed towards a veterinary audience, we will discuss the impact of WNV infection in horses and humans, historical efforts and methods used to control WNV, and the potential of recently developed genetic techniques that target the immune system of the mosquito as a tool in reducing the transmission of WNV.

### 1.1. Transmission Cycle

The transmission cycle of WNV involves birds as a reservoir and amplifying host, mosquitoes as the transmitting vector of WNV, and mammals serving as dead-end hosts that exhibit disease. Many species of birds are capable of functioning as a reservoir, with the American robin considered the main host species [1]. Mosquitoes become infected with WNV when a blood meal is ingested from a viremic bird, and the virus then replicates within mosquito cells and accumulates in the salivary glands, which facilitates transmission of the virus via saliva when the mosquito next feeds [1].

Generally, humans and other mammals do not achieve a viremia significant enough to facilitate transmission of WNV to mosquitoes and are thus considered dead-end hosts [5]. A few exceptions to this rule have been found through experimental infection. Immunosuppressed mammals and some small mammals like rodents and rabbits can achieve a viremia significant enough to infect mosquitoes, although it is not known whether mammal-to-mosquito transmission contributes significantly to the transmission cycle of WNV [6]. Iatrogenic transmission via blood transfusion or organ transplantation as well as vertical transmission have been reported in humans, but both iatrogenic and vertical transmission are thought to be uncommon [1]. In the United States, donated blood products and some organs are routinely screened for WNV prior to use [3]. Recent systematic reviews have shown that environmental factors and climate change are important influencers of WNV distribution, transmission, and mosquito habitat [7,8].

### 1.2. Disease Manifestation

Disease caused by West Nile virus is most significant in humans and equids, and there are many commonalities when comparing epidemiology and manifestations of disease between these species. In both humans and equines, 80% of infected individuals are asymptomatic [9]. Humans who are clinical following WNV infection most commonly report symptoms of fever, fatigue, and muscle aches, while most horses clinical for the disease display signs of fever and lethargy [5,9]. In the United States, West Nile virus is a reportable disease in humans as well as equines, and clinicians who suspect WNV infection must contact public health authorities [9].

In both humans and horses, a small portion (less than 1% in humans) of clinically affected individuals will develop neurologic signs due to the virus causing meningoencephalitis, inflammation of the brain and meninges. Neurologic signs seen in both species due to WNV infection include ataxia, blindness, paralysis, and muscle fasciculation. In humans who display neurologic signs, fatality rates of up to 20% have been reported. In horses, up to 30% of individuals with neurologic signs die, with an additional 10–20% experiencing neurologic deficits following recovery [9]. A recent review compares the similarities between West Nile disease in humans and horses [10].

### 1.3. Diagnosis and Treatment

Diagnosis of West Nile virus infection is made similarly in both humans and equines, through a combination of compatible clinical signs and laboratory testing. The clinical signs needed to diagnose a horse with WNV vary from country to country. In Canada, the compatible clinical signs must include ataxia plus two or more of the following: circling, hind limb weakness, inability to stand, multiple limb paralysis, muscle fasciculation, proprioceptive deficits, blindness, lip droop/paralysis, teeth grinding, fever, and acute death [11]. A horse that displays these signs and has a positive confirmatory laboratory test is diagnosed with WNV infection [9]. The most common confirmatory laboratory test is IgM ELISA of serum or cerebrospinal fluid (CSF), although molecular biology techniques such as polymerase chain reaction (PCR), which detects viral RNA, or immunohistochemistry (IHC), which uses antibodies that target virus-associated antigen, can also be used [9].

Treatment of WNV is purely symptomatic; no specific treatments exist in either humans or equines, so care of infected patients focuses on managing the symptoms caused by the virus [1]. Principles of treatment in both species include maintaining adequate hydration and managing inflammation-associated pain. In patients with severe neurologic symptoms, seizure control and ventilatory support are often required [3]. Antunes de Castro-Jorge et al. reviewed clinical presentation, diagnosis, treatment, and prophylaxis of WNV infections in horses and humans [12].

## 2. Vaccines against West Nile virus

### 2.1. Equine Vaccination

The lack of any specific treatment options heightens the importance of preventative measures in the management of West Nile virus. Two strategies of prevention currently exist: vaccination and mosquito control.

Vaccine development has been a major focus of the response to WNV since the emergence of the disease. In horses, efforts have resulted in four USDA licensed vaccines, with WNV considered a core vaccination by the American Association of Equine Practitioners (AAEP). Core vaccines protect from diseases with potential public health significance that are endemic to a region. Core vaccines protect against virulent or highly infectious pathogens, reducing the risk of severe disease. Core vaccines are efficacious and safe, thus exhibiting a high level of patient benefit and low level of risk, justifying their use in patients. Currently available WNV vaccines include two killed whole-virus vaccines, a modified live recombinant canary pox vaccine containing WNV pre-membrane and envelope genes and a chimera vaccine that combines WNV antigen with inactivated flavivirus [9,13] (Table 1). All of the available vaccines generate protective immunity and have been licensed on the basis of fulfilling one or more of the following criteria: (1) aiding in the prevention of viremia, (2) aiding in the reduction in viremia, encephalitis, and clinical disease, (3) aiding in the prevention of disease, viremia, and encephalitis, and (4) aiding in the prevention of viremia and mortality, while also aiding in the reduction in severity of clinical disease. All currently licensed WNV vaccines are administered via intramuscular injection. Following an initial primary series, these vaccines are administered annually, although they can be administered more frequently under special circumstances, such as in horses with reduced immune function like geriatrics or horses treated with corticosteroids long term [13]. Equine WNV vaccinations are an effective tool, but their existence and use do not preclude the need for other control measures.

### 2.2. Bird Vaccination

WNV transmission cycles through birds that act as an amplifying host [14]. The virus replicates to high titers in birds, and they have high WNV viremia compared to mammals [15]. Thus, mosquitoes can become infected when they take a blood meal from WNV-infected birds but not from mammals that display low viremia. However, mosquitoes can reinfect the bird population, continuing enzootic infection. Furthermore, bird migration allows for the distribution of WNV beyond the mosquito range [16]. Like mammals, WNV-infected birds generate neutralizing antibodies that provide long-lasting protection over the course of multiple WNV seasons [17,18]. Additionally, chicks inherit maternally derived WNV antibodies [19]. Considering the importance of birds in the WNV transmission cycle and their ability to generate neutralizing antibodies, there has been considerable effort to develop vaccination strategies in birds [20].

Bird vaccination would be especially important during WNV outbreaks. During this time, vaccines could be administered in captive birds, breeding centers, and birds released into the wild for restocking purposes and surveillance programs [21]. Vaccine design for birds has been based on successful vaccination programs used in horses. A chimeric virus using yellow fever virus expressing WNV surface proteins was tested in crows [22]. Vaccination with WNV subviral particles provided increased survival rates in magpies challenged with WNV [21,23]. DNA-based vaccines expressing prM/E were tested in condors and falcons and provided protective immunity [24,25]. These vaccination regimens, among many others, were tested in both wild and domestic birds [20]. While the vaccination regimens tested provided protection, sterilizing immunity was not achieved since virus replication was still observed in vaccinated birds. Nevertheless, the levels of viremia were sufficiently decreased to a point that would not allow mosquitoes to become infected after taking a blood meal from these vaccinated and challenged birds [14,21,24]. Taken together, efforts to develop and implement bird vaccination should continue.

### 2.3. Human Vaccination

Currently, no West Nile virus vaccine has been approved for human use (Table 1). Numerous candidates of various types have entered clinical trials, although none has advanced past phase II of the FDA’s four-phase licensure process [26]. The WNV vaccine developed by the Vaccine Research Center (VRC) expressing pre-membrane (prM) and envelope (E) glycoprotein reached stage I clinical trials and showed the presence of neutralizing antibodies in over 90% of young and old human subjects after receiving three doses [27]. Also expressing prM and E is a recombinant yellow fever vaccine that generated neutralizing antibodies in 90% of young and old human subjects after only one dose [28]. This vaccine moved into phase II clinical trials. Expressing prM and E using attenuated DENV provided neutralizing antibodies in 89% of subjects after two doses but only reached phase I clinical trials [29]. A recombinant subunit vaccine expressing truncated E (HBV-002) provided neutralizing antibodies in all subjects following three doses but also only reached phase I clinical trials [30]. Two vaccines utilize inactivated/killed virus. Hydrogen peroxide-inactivated WNV provided neutralizing antibodies in half of the human subjects after two doses [31], and formaldehyde inactivation also generated neutralizing antibodies [32]. These vaccines progressed to phase I/II clinical trials. Together, these data from phase I and II trials of WNV vaccine candidates show promising efficacy and no safety concerns [26,33]. However, the obstacles that have thus far impeded development of a human WNV vaccine lie outside the realms of vaccine efficacy and safety. One such challenge involves the logistics of the FDA’s licensure process and the epidemiology of WNV. Phase III clinical trials require several thousand trial participants, a significant increase in sample size from phases I (between 10 and 100 participants) and II (several hundred participants) [34]. West Nile virus causes sporadic disease outbreaks with high regional variability, making it logistically challenging to coordinate a clinical trial on the scale required for phase III of the FDA process [26].

For a human WNV vaccine to reach FDA licensure, an alternate pathway to licensure would likely be required [26]. There is precedence for vaccinations utilizing alternate pathways, such as via animal models. However, the use of animal models highlights another challenge. A hypothetical alternative phase III trial utilizing non-human primates as a model for humans would need to establish a threshold for neutralizing antibodies that would function as a correlate for protection, which is an indicator of protective immunity. Such an approach was utilized in research with yellow fever virus, another member of the flavivirus genus. In contrast to yellow fever virus, non-human primates are not very susceptible to WNV, meaning the establishment of a neutralizing antibody threshold is difficult. An additional challenge related to the establishment of a correlate for protection is the fact that most individuals infected with WNV are asymptomatic and, thus, a prospective study might require an even larger sample size to obtain enough individuals with symptoms for the determination of the antibody threshold [26].

Additionally, an alternative pathway to FDA licensure requires that a manufacturer demonstrates the socioeconomic benefit of their vaccine, along with the clinical benefit [26]. Published studies focusing on the cost-effectiveness of WNV vaccination in the United States concluded that universal vaccination would not result in reduced cost to society at-large as compared to the cost of not developing a vaccine and treating the larger number of affected individuals resulting from an unvaccinated population [35,36]. An immunization strategy that targets only those groups most at-risk would be more cost-effective but does not compare favorably with other currently licensed vaccines [36]. Currently, a human WNV vaccination is not cost-effective; a drop in research and manufacturing costs, an increase in WNV case numbers, or the emergence of a new genetic variant that is more likely to cause severe neurologic disease would need to occur for human WNV vaccination to be economically advantageous on a societal level [35].

## 3. Mosquito Control

### 3.1. Conventional Mosquito Control Methods

Since the first outbreak of West Nile virus in the United States, which occurred in New York City in 1999, mosquito population control has been a mainstay of the response to West Nile virus [37]. Initial mosquito control programs were reactive in response to the high case numbers of illness seen in mammals. Reactive measures, while effective, are expensive. The response to the New York state 1999 outbreak was estimated to cost over $14 million, but this does not account for the initial morbidity caused by the virus, which allowed time for significant spread. The development of robust surveillance and data sharing systems has facilitated the implementation of proactive control strategies that attempt to prevent outbreaks rather than abate them. Surveillance strategies like monitoring case numbers in birds (i.e., using them as a sentinel species) and directly monitoring trends in mosquito populations allow for targeted control measures prior to the occurrence of outbreaks in mammals [37]. Modern integrated vector management (IVM) programs combine proactive and reactive approaches with public education programs to maximize efficiency, along with increasingly complex surveillance programs that account for ecological factors, weather, vector dynamics, and mammalian demographic factors [38].

Specific techniques used in IVM programs are divided into three categories: source control, larvae control, and adult control [38]. Source control techniques reduce the habitable environment for both larvae and adults while larvae control and adult control target their respective life stages through measures like pesticide application, introduction of predatory species, and trapping. For example, there are increased mosquito populations after winter flooding of farmland [39], when sewage outflow is increased [40] in urban areas [41], and in wetlands enriched with ammonium nitrogen. The use of small crustaceans provides biological control of larval populations of *Culex quinquefasciatus* in natural water-containing ditches, and pollution is detrimental to these natural copepod predators of mosquito larvae [42]. Each of these techniques varies in scale, from population-level measures that aim to affect a broad geographic area (e.g., a municipal government applying pesticides to public lands) to personal protective measures (e.g., an individual remaining indoors during times of day when mosquitoes are most active). High variability in the techniques employed across localities exists, influenced by factors such as cost, public perception, practical viability, ecological impact, and legality [38]. Measuring the effectiveness of proactive control programs is currently challenging and an area of active research. Reactive control programs are generally regarded as effective. For example, one study undertaken in Sacramento County, California, during a WNV outbreak reported a decrease in the mosquito infection rate from 8.2/1000 to 4.3/1000 in areas treated with the adulticide pyrethrin while the infection rate climbed from 2.0/1000 to 8.7/1000 in untreated areas over the same period [37].

### 3.2. Genetic Mosquito Control Methods

In considering what an ideal new tool against WNV infection might look like, several criteria stand out. Chiefly, a technique that can address WNV in humans and horses, is broadly deployable, can be utilized proactively, and avoids the ecological pitfalls present with some conventional mosquito control measures (i.e., development of pesticide resistance, environmental disruption caused by water management techniques) would be especially desirable. It is for these reasons that genetic mosquito control methods have gained attention. These methods directly address the vector of WNV, do not require an existing disease outbreak to be effective, and have minimal impact on other species [43].

Enabled by research that has increased human understanding of the mosquito immune system, and specifically the mosquito immune response to WNV, several techniques that modify the mosquito immune response to WNV are under active research and investigation [5,43,44]. Broadly, these techniques fall into one of two categories: (1) transgenic techniques, which directly modify the mosquito immune response, and (2) paratransgenic techniques, which utilize a symbiotic organism, such as a bacteria, to affect the mosquito immune response [44].

Transgenic techniques can be further subcategorized into refractory mosquito techniques and lethal gene techniques [43]. Refractory mosquito techniques involve the introduction of a target gene and a gene drive into a mosquito. The target gene is a gene involved in the mosquito immune response to WNV, and this target gene is either upregulated or downregulated to enhance the immune response. The goal is to result in a lower viral load and reduced transmission of WNV. The gene drive facilitates the spread of the target gene to progeny of the mosquito, which means a refractory mosquito system is self-sustaining. In an ideal outcome, a low number of releases of genetically modified mosquitoes would be sufficient to increase the frequency of the target gene, eventually driving it to fixation within a population [43].

While the refractory mosquito technique is conceptually promising, the challenges to its development and implementation lie in its complexity. It requires the identification of a target gene, development of a technique able to modify the target gene, identification of a gene drive system compatible with the target gene, and use of a system that can insert both the target gene and the gene drive into the mosquito genome. The resulting transgenic mosquito must also have comparable genetic fitness to wild-type mosquitoes for the target gene to fix in the population. In addition, transgenic strains are species-specific, meaning each mosquito species would require its own transgenic refractory system [43].

Lethal gene techniques introduce a dominant lethal gene into the mosquito genome. A female-specific gene promoter is paired with the lethal gene, which causes the gene to only be expressed in females but carried in males. The genetically modified males, upon mating with wild-type females, will produce nonviable female progeny and viable male progeny [15]. Over time, this results in a decline in the mosquito population. Unlike the refractory mosquito technique, the lethal gene technique is not self-sustaining and requires a greater number of releases of genetically modified mosquitoes to produce the desired result. The lethal gene techniques share some of the same challenges to implementation as the refractory system, namely, the potential effect on mosquito fitness and the need for species-specific transgenic strains [45]. Lethal gene techniques have been used in several countries to combat dengue viruses via transgenic strains of *Aedes aegypti*, so there is precedence for their real-world use, although further research into the development of transgenic *Culex* is necessary in order to produce a transgenic strain suitable for use [43].

With a paratransgenic approach, a symbiotic organism that survives within the mosquito, usually bacteria, is identified [44]. Simultaneously, an effector molecule that modifies the mosquito immune response in the desired way is identified. Finally, the symbiotic organism is genetically modified to produce the effector molecule. Depending on the effector molecule selected, a paratransgenic system can either be population suppressing, like the transgenic lethal gene system, or refractory, like the transgenic refractory mosquito system. The organism is then fed to mosquitoes in a lab setting, and the lab-reared mosquitoes are released into the environment to spread the symbiotic organism. Since the mosquito itself is not transgenic, there is no effect on fitness, and the paratransgenic mosquitoes have identical reproductive fitness to wild-type mosquitoes. A second advantage over transgenic systems is the ability of a paratransgenic system to be utilized in multiple mosquito species, making it particularly well suited to areas where multiple species of mosquito transmit WNV. Additionally, the genetic engineering of bacteria is simpler and more well understood than the production of transgenic mosquitoes, making paratransgenesis a more realistic approach [44].

Challenges inherent to paratransgenic techniques include the cultivation of symbiotic organisms for study since the conditions these organisms thrive under are difficult to recreate in a laboratory, although ribosomal RNA sequencing is a potential alternative to laboratory cultivation that would allow for the necessary studies [44]. Thorough study of potential symbiotic organisms is critical as the presence of a wild-type symbiotic organism can affect the mosquito immune response to WNV. One study found the presence of the symbiotic bacteria *Wolbachia* increased WNV titer in *Culex tarsalis* while other studies correlated the presence of *Wolbachia* with inhibition of WNV in other *Culex* species [5]. Interactions between the symbiotic organism and the species of interest must be understood prior to use in a paratransgenic system as the symbiotic organism itself may have undesirable effects. However, new approaches for WNV are imperative since current strategies that use *Wolbachia* to reduce viral infection in mosquitoes [46,47,48] have been shown to enhance WNV infection [49].

## 4. The Complexity of Responding to WNV and the Swiss Cheese Model

Conventional mosquito control techniques and the equine WNV vaccine are effective tools in the response to WNV, but disease outbreaks remain a continued problem. From a One Health perspective, the challenge of West Nile virus is complex, involving numerous animal species and many environmental factors. The response has been equally complex, and it has become apparent that many effective tools and techniques, used in combination, are needed to address the challenge.

**Figure 1:**
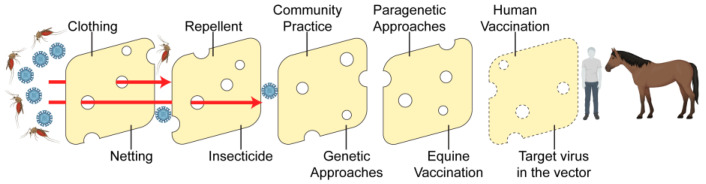
The Swiss cheese model as applied to West Nile virus prevention. Solid “slices” represent currently used techniques, dotted “slices” represent emerging or hypothetical techniques. Figure created using Biorender, (https://www.biorender.com (accessed on 27 April 2024)) adapted from Ian M. Mackay.

The response to WNV lends itself well to the Swiss cheese model, in which the various effective prevention strategies each have varied areas of strength and weakness, like individual slices of Swiss cheese have solid areas and holes, and the combination of these strategies creates a more effective response than any strategy used individually (Figure 1). Originally developed by James Reason to explain systems’ failures, the Swiss cheese model gained popularity in the field of epidemiology when applied to the COVID-19 pandemic [50]. When applied to WNV, novel techniques, such as a hypothetical human vaccine or new mosquito control methods, can be thought of as an additional slice added to the already existing set of slices that decrease transmission of WNV to mammals. For example, clothing, netting, and mosquito repellent act as physical barriers to mosquito bites and potential WNV infection. Insecticides containing plant-derived pyrethrins can specifically target adult mosquitoes and be used to reduce WNV infection [51]. Best practices at the individual and community levels can also reduce mosquito populations. Emptying standing water and reducing light pollution can reduce the risk of infection [52]. Combining these approaches with the genetic and vaccination strategies described above will reduce overall infection rates. Further progress towards human vaccination will add another barrier. Finally, recent work shows that levels of virus can be reduced within the mosquito by targeting insulin/insulin-like growth factor signaling (IIS). Provisioning *Culex* mosquitoes with bovine insulin in their blood meal reduced WNV levels via an Akt-mediated, JAK/STAT-dependent, RNAi-independent mechanism [53]. However, the simultaneous activation of JAK/STAT- and RNAi-mediated immunity in *Aedes* mosquitoes using small molecules reduced the prevalence and levels of Zika virus in these mosquitoes [54]. A similar approach could be used in *Culex* mosquitoes to combat WNV infection. Bait-delivered small molecules that activate these antiviral pathways in mosquitoes could form the basis of future strategies to reduce virus transmission. Taken together, an IIS-targeted approach could be combined with physical, behavioral, and ecological barriers already in use for arbovirus transmission control as well as transgenic and paratransgenic techniques. Several combined layers of protection against arbovirus transmission will significantly reduce overall infection risk.

## 5. Conclusions and Future Directions

West Nile virus continues to be a disease of concern to both human and veterinary health. Additionally, there are occupational risks associated with WNV infections [55,56]. Odigie et al. found that military workers, veterinarians, agricultural workers, farmers, and laboratory workers were at higher risk for WNV infections due to increased contact with infected fluids and aerosols. They concluded that the identification of these workers could increase surveillance of them by physicians and improve preventative measures [57]. Major advances in preventative strategies, to include equine vaccinations and increasingly sophisticated monitoring and mosquito control methods, have been developed since the disease first appeared in the United States in 1999. However, a need still exists for novel solutions, especially targeted strategies that impact the vector mosquito without damaging the environment or other species. Genetic methods of mosquito control are an attractive option as they simultaneously address WNV in both humans and equines. However, there are a number of practical, ethical, and regulatory challenges to genetic methods of controlling mosquito-borne infections [58,59]. For example, biosafety approval is needed, and these regulations may vary for different countries. Policies regarding the transport of genetically modified mosquitoes both intentionally or by natural migration need to be considered [59]. For example, in the United States, the Food and Drug Administration assessed the risk to public health and the ecosystem before a field trial of genetically modified *Aedes aegypti* in Key Haven, Florida, was allowed [58]. Nevertheless, methods to genetically engineer mosquitoes and conduct field trials are an area of active research and development, although conceptually very promising. Both transgenic and paratransgenic approaches have features that make them desirable and particularly well suited to WNV management in certain areas as well as challenges to their development and implementation.

## Figures and Tables

**Table 1:** West Nile virus vaccination types and licensure.

Vaccine Name	Type	Animal	Licensed?
West Nile-Innovator	Killed virus vaccine	Horse	Yes
Prestige WNV	Killed virus vaccine	Horse	Yes
Recombitek WNV	Recombinant canary pox vaccine	Horse	Yes
PreveNile	Inactivated flavivirus chimera vaccine	Horse	Yes
WNV RSP	Recombinant subviral particles (RSP) purified from Hela cells	Bird	No
ChimeriVax-WN	Yellow fever vaccine strain 17D expressing pre-membrane and E glycoprotein (prM/E)	Bird	No
WNV-DNA-1/2	Recombinant vaccine expressing E	Bird	No
pVWN	DNA vaccine expressing prM/E	Bird	No
VRC WNV	DNA vaccine expressing prM/E	Human	No
ChimeraVax-WN02	Recombinant yellow fever vaccine expressing prM/E	Human	No
HBV-002	Recombinant subunit vaccine expressing truncated E	Human	No
rWN/DEN4 30	Recombinant DENV expressing prM/E	Human	No
Hydrovax-001	H_2_O_2_-killed virus vaccine	Human	No
Inactivated WNV	Formaldehyde-killed virus vaccine	Human	No

## References

[1] Colpitts T.M., Conway M.J., Montgomery R.R., Fikrig E. (2012). West Nile Virus: Biology, transmission, and human infection. Clin. Microbiol. Rev..

[2] Orba Y., Hang’ombe B.M., Mweene A.S., Wada Y., Anindita P.D., Phongphaew W., Qiu Y., Kajihara M., Mori-Kajihara A., Eto Y. (2018). First isolation of West Nile virus in Zambia from mosquitoes. Transbound. Emerg. Dis..

[3] Hadler J.L., Patel D., Bradley K., Hughes J.M., Blackmore C., Etkind P., Kan L., Getchell J., Blumenstock J., Engel J. (2014). National capacity for surveillance, prevention, and control of West Nile virus and other arbovirus infections--United States, 2004 and 2012. MMWR Morb. Mortal. Wkly. Rep..

[4] Miller M.J., Loaiza J.R. (2015). Geographic expansion of the invasive mosquito Aedes albopictus across Panama--implications for control of dengue and Chikungunya viruses. PLoS Neglected Trop. Dis..

[5] Ahlers L.R.H., Goodman A.G. (2018). The Immune Responses of the Animal Hosts of West Nile Virus: A Comparison of Insects, Birds, and Mammals. Front. Cell. Infect. Microbiol..

[6] Bowen R.A., Nemeth N.M. (2007). Experimental infections with West Nile virus. Curr. Opin. Infect. Dis..

[7] Giesen C., Herrador Z., Fernandez-Martinez B., Figuerola J., Gangoso L., Vazquez A., Gómez-Barroso D. (2023). A systematic review of environmental factors related to WNV circulation in European and Mediterranean countries. One Health.

[8] D’Amore C., Grimaldi P., Ascione T., Conti V., Sellitto C., Franci G., Kafil S.H., Pagliano P. (2022). West Nile Virus diffusion in temperate regions and climate change. A systematic review. Infez Med..

[9] Paré J., Moore A. (2018). West Nile virus in horses—What do you need to know to diagnose the disease?. Can. Vet. J..

[10] Schwarz E.R., Long M.T. (2023). Comparison of West Nile Virus Disease in Humans and Horses: Exploiting Similarities for Enhancing Syndromic Surveillance. Viruses.

[11] Government of Canada West Nile Virus: Surveillance. https://inspection.canada.ca/animal-health/terrestrial-animals/diseases/immediately-notifiable/west-nile-virus/surveillance/eng/1346131121021/1346131213336.

[12] Castro-Jorge L.A., Siconelli M.J.L., Ribeiro B.D.S., Moraes F.M., Moraes J.B., Agostinho M.R., Klein T.M., Floriano V.G., Fonseca B. (2019). West Nile virus infections are here! Are we prepared to face another flavivirus epidemic?. Rev. Soc. Bras. Med. Trop..

[13] Desanti-Consoli H., Bouillon J., Chapuis R.J.J. (2022). Equids’ Core Vaccines Guidelines in North America: Considerations and Prospective. Vaccines.

[14] Komar N., Langevin S., Hinten S., Nemeth N., Edwards E., Hettler D., Davis B., Bowen R., Bunning M. (2003). Experimental infection of North American birds with the New York 1999 strain of West Nile virus. Emerg. Infect. Dis..

[15] Kramer L.D., Bernard K.A. (2001). West Nile virus infection in birds and mammals. Ann. N. Y. Acad. Sci..

[16] Reed K.D., Meece J.K., Henkel J.S., Shukla S.K. (2003). Birds, migration and emerging zoonoses: West nile virus, lyme disease, influenza A and enteropathogens. Clin. Med. Res..

[17] Nemeth N.M., Kratz G.E., Bates R., Scherpelz J.A., Bowen R.A., Komar N. (2008). Naturally induced humoral immunity to West Nile virus infection in raptors. Ecohealth.

[18] Nemeth N.M., Oesterle P.T., Bowen R.A. (2009). Humoral immunity to West Nile virus is long-lasting and protective in the house sparrow (Passer domesticus). Am. J. Trop. Med. Hyg..

[19] Baitchman E.J., Tlusty M.F., Murphy H.W. (2007). Passive transfer of maternal antibodies to West Nile virus in flamingo chicks (Phoenicopterus chilensis and Phoenicopterus ruber ruber). J. Zoo Wildl. Med..

[20] deOya N.J., Escribano-Romero E., Blazquez A.B., Martin-Acebes M.A., Saiz J.C. (2019). Current Progress of Avian Vaccines Against West Nile Virus. Vaccines.

[21] deOya N.J., Escribano-Romero E., Camacho M.C., Blazquez A.B., Martín-Acebes M.A., Höfle U., Saiz J.C. (2019). A Recombinant Subviral Particle-Based Vaccine Protects Magpie (Pica pica) Against West Nile Virus Infection. Front. Microbiol..

[22] Langevin S.A., Arroyo J., Monath T.P., Komar N. (2003). Host-range restriction of chimeric yellow fever-West Nile vaccine in fish crows (Corvus ossifragus). Am. J. Trop. Med. Hyg..

[23] Merino-Ramos T., Blázquez A.B., Escribano-Romero E., Cañas-Arranz R., Sobrino F., Saiz J.C., Martín-Acebes M.A. (2014). Protection of a single dose west nile virus recombinant subviral particle vaccine against lineage 1 or 2 strains and analysis of the cross-reactivity with Usutu virus. PLoS ONE.

[24] Fischer D., Angenvoort J., Ziegler U., Fast C., Maier K., Chabierski S., Eiden M., Ulbert S., Groschup M.H., Lierz M. (2015). DNA vaccines encoding the envelope protein of West Nile virus lineages 1 or 2 administered intramuscularly, via electroporation and with recombinant virus protein induce partial protection in large falcons (*Falco* spp.). Vet. Res..

[25] Chang G.J., Davis B.S., Stringfield C., Lutz C. (2007). Prospective immunization of the endangered California condors (Gymnogyps californianus) protects this species from lethal West Nile virus infection. Vaccine.

[26] Ulbert S. (2019). West Nile virus vaccines—Current situation and future directions. Hum. Vaccin. Immunother..

[27] Ledgerwood J.E., Pierson T.C., Hubka S.A., Desai N., Rucker S., Gordon I.J., Enama M.E., Nelson S., Nason M., Gu W. (2011). A West Nile virus DNA vaccine utilizing a modified promoter induces neutralizing antibody in younger and older healthy adults in a phase I clinical trial. J. Infect. Dis..

[28] Dayan G.H., Pugachev K., Bevilacqua J., Lang J., Monath T.P. (2013). Preclinical and clinical development of a YFV 17 D-based chimeric vaccine against West Nile virus. Viruses.

[29] Durbin A.P., Wright P.F., Cox A., Kagucia W., Elwood D., Henderson S., Wanionek K., Speicher J., Whitehead S.S., Pletnev A.G. (2013). The live attenuated chimeric vaccine rWN/DEN4Δ30 is well-tolerated and immunogenic in healthy flavivirus-naïve adult volunteers. Vaccine.

[30] Lieberman M.M., Nerurkar V.R., Luo H., Cropp B., Carrion R., de la Garza M., Coller B.A., Clements D., Ogata S., Wong T. (2009). Immunogenicity and protective efficacy of a recombinant subunit West Nile virus vaccine in rhesus monkeys. Clin Vaccine Immunol..

[31] Woods C.W., Sanchez A.M., Swamy G.K., McClain M.T., Harrington L., Freeman D., Poore E.A., Slifka D.K., Poer DeRaad D.E., Amanna I.J. (2019). An observer blinded, randomized, placebo-controlled, phase I dose escalation trial to evaluate the safety and immunogenicity of an inactivated West Nile virus Vaccine, HydroVax-001, in healthy adults. Vaccine.

[32] Barrett P.N., Terpening S.J., Snow D., Cobb R.R., Kistner O. (2017). Vero cell technology for rapid development of inactivated whole virus vaccines for emerging viral diseases. Expert Rev. Vaccines.

[33] Gould C.V., Staples J.E., Huang C.Y., Brault A.C., Nett R.J. (2023). Combating West Nile Virus Disease—Time to Revisit Vaccination. N. Engl. J. Med..

[34] Chen R.T., Shimabukuro T.T., Martin D.B., Zuber P.L., Weibel D.M., Sturkenboom M. (2015). Enhancing vaccine safety capacity globally: A lifecycle perspective. Vaccine.

[35] Zohrabian A., Hayes E.B., Petersen L.R. (2006). Cost-effectiveness of West Nile virus vaccination. Emerg. Infect. Dis..

[36] Shankar M.B., Staples J.E., Meltzer M.I., Fischer M. (2017). Cost effectiveness of a targeted age-based West Nile virus vaccination program. Vaccine.

[37] Nasci R.S., Mutebi J.P. (2019). Reducing West Nile Virus Risk Through Vector Management. J. Med. Entomol..

[38] Bellini R., Zeller H., Van Bortel W. (2014). A review of the vector management methods to prevent and control outbreaks of West Nile virus infection and the challenge for Europe. Parasit Vectors.

[39] Lawler S.P., Dritz D.A. (2006). Effects of rice straw and water management on riceland mosquitoes. J. Med. Entomol..

[40] Sanford M.R., Chan K., Walton W.E. (2005). Effects of inorganic nitrogen enrichment on mosquitoes (Diptera: Culicidae) and the associated aquatic community in constructed treatment wetlands. J. Med. Entomol..

[41] Chaves L.F., Keogh C.L., Vazquez-Prokopec G.M., Kitron U.D. (2009). Combined sewage overflow enhances oviposition of Culex quinquefasciatus (Diptera: Culicidae) in urban areas. J. Med. Entomol..

[42] Marten G.G., Nguyen M., Mason B.J., Ngo G. (2000). Natural control of Culex quinquefasciatus larvae in residential ditches by the copepod Macrocyclops albidus. J. Vector Ecol..

[43] Wilke A.B.B., Beier J.C., Benelli G. (2018). Transgenic Mosquitoes—Fact or Fiction?. Trends Parasitol..

[44] Wilke A.B., Marrelli M.T. (2015). Paratransgenesis: A promising new strategy for mosquito vector control. Parasit Vectors.

[45] Coleman P.G., Alphey L. (2004). Genetic control of vector populations: An imminent prospect. Trop. Med. Int. Health.

[46] Thomas S., Verma J., Woolfit M., O’Neill S.L. (2018). Wolbachia-mediated virus blocking in mosquito cells is dependent on XRN1-mediated viral RNA degradation and influenced by viral replication rate. PLoS Pathog..

[47] Dodson B.L., Andrews E.S., Turell M.J., Rasgon J.L. (2017). Wolbachia effects on Rift Valley fever virus infection in Culex tarsalis mosquitoes. PLoS Neglected Trop. Dis..

[48] Koh C., Audsley M.D., Di Giallonardo F., Kerton E.J., Young P.R., Holmes E.C., McGraw E.A. (2019). Sustained Wolbachia-mediated blocking of dengue virus isolates following serial passage in Aedes aegypti cell culture. Virus Evol..

[49] Dodson B.L., Hughes G.L., Paul O., Matacchiero A.C., Kramer L.D., Rasgon J.L. (2014). Wolbachia enhances West Nile virus (WNV) infection in the mosquito Culex tarsalis. PLoS Neglected Trop. Dis..

[50] Ngo T. (2021). To slow the spread of COVID-19, we need to bring back the Swiss Cheese Model of pandemic response. Health Aff. Forefr..

[51] Shapiro H., Micucci S. (2003). Pesticide use for West Nile virus. CMAJ.

[52] Coetzee B.W.T., Gaston K.J., Koekemoer L.L., Kruger T., Riddin M.A., Smit I.P.J. (2022). Artificial Light as a Modulator of Mosquito-Borne Disease Risk. Front. Ecol. Evol..

[53] Ahlers L.R.H., Trammell C.E., Carrell G.F., Mackinnon S., Torrevillas B.K., Chow C.Y., Luckhart S., Goodman A.G. (2019). Insulin Potentiates JAK/STAT Signaling to Broadly Inhibit Flavivirus Replication in Insect Vectors. Cell Rep..

[54] Trammell C.E., Ramirez G., Sanchez-Vargas I., St Clair L.A., Ratnayake O.C., Luckhart S., Perera R., Goodman A.G. (2022). Coupled small molecules target RNA interference and JAK/STAT signaling to reduce Zika virus infection in Aedes aegypti. PLoS Pathog..

[55] Chirico F., Magnavita N. (2019). The West Nile virus epidemic-occupational insight. Lancet.

[56] Burki T. (2018). Increase of West Nile virus cases in Europe for 2018. Lancet.

[57] Odigie A.E., Stufano A., Schino V., Zarea A.A.K., Ndiana L.A., Mrenoshki D., Ugochukwu I.C.I., Lovreglio P., Greco G., Pratelli A. (2024). West Nile Virus Infection in Occupational Settings-A Systematic Review. Pathogens.

[58] Meghani Z. (2022). Regulation of genetically engineered (GE) mosquitoes as a public health tool: A public health ethics analysis. Glob. Health.

[59] James S.L., Dass B., Quemada H. (2023). Regulatory and policy considerations for the implementation of gene drive-modified mosquitoes to prevent malaria transmission. Transgenic Res..

